# Hsa_circ_0060467 promotes breast cancer liver metastasis by complexing with eIF4A3 and sponging miR-1205

**DOI:** 10.1038/s41420-023-01448-4

**Published:** 2023-05-09

**Authors:** Yan Zeng, Wei Du, Zhongying Huang, Song Wu, Xueqi Ou, Jinhui Zhang, Cheng Peng, Xiaoqing Sun, Hailin Tang

**Affiliations:** 1 State Key Laboratory of Oncology in South China, Sun Yat-sen University Cancer Center, Guangzhou, Guangdong China; 2 The Fifth Affiliated Hospital of Sun Yat-sen University, Zhuhai, Guangdong China; 3 Department of Pathology, the First People’s Hospital of Changde City, Changde, Hunan China; 4 State Key Laboratory of Southwestern Chinese Medicine Resources, Chengdu University of Traditional Chinese Medicine, Chengdu, Sichuan China

**Keywords:** Breast cancer, Metastasis

## Abstract

Breast cancer (BC) is the most common cancer and the top cause of female mortality worldwide. The prognosis for patients with breast cancer liver metastasis (BCLM) remains poor. Emerging studies suggest that circular RNAs (circRNAs) are associated with the progression of BC. Exploration of circRNAs presents a promising avenue for identifying metastasis-targeting agents and improving the prognosis of patients with BCLM. Microarray and bioinformatic analyses were used to analyze differentially expressed circRNAs between three pairs of BCLM and primary BC. The roles of hsa_circ_0060467 (circMYBL2) and its target gene E2F1 in BC cells were explored by multiple functional experiments. And xenograft mouse models and hepatic metastases of BC hemi-spleen models were used to illustrate the function of circMYBL2 in vivo. The intrinsic molecular mechanism involving circMYBL2 was confirmed by bioinformatics analyses, RIP assays, CHIRP assays, luciferase reporter assays, and rescue experiments. CircMYBL2 was overexpressed in BCLM tissues and BC cells. Functionally, circMYBL2 can facilitate the proliferation and liver metastasis of BC. Mechanistically, circMYBL2 upregulated the transcription factor E2F1 by sponging miR-1205 and complexing with eukaryotic translation initiation factor 4A3 (eIF4A3) and then facilitated the epithelial-mesenchymal transition (EMT) process in BC cells. Our findings showed that circMYBL2 promoted the tumorigenesis and aggressiveness of BC through the circMYBL2/miR-1205/E2F1 and circMYBL2/eIF4A3/E2F1 axes, which may provide a novel targeted therapy for patients with BCLM.

## Introduction

According to an evaluation in 2020, breast cancer (BC) is the most common tumor worldwide. A total of 2.3 million new cases are estimated to be diagnosed, comprising 24.5 percent of female cancer cases. This disease has the highest mortality, with greater than 680 000 deaths, representing ~15.5% of female cancer deaths [1]. It has been reported that even after diagnosis and primary tumor therapy, 20–30% of BC patients may develop metastasis, and metastatic cancer results in 90% cancer-related mortality [2, 3]. The liver ranks as the third most common organ in BC metastasis. However, the long-term survival rate of patients suffering from breast cancer liver metastasis (BCLM) is projected to be 8.5% (5 years), which is poorer than that of patients with locoregional metastasis, bone metastasis, or lung relapse [2, 4]. Therefore, systematic and comprehensive research concerning the BCLM molecular mechanism should be performed to discover metastasis-targeting agents and improve its prognosis. The liver, with its abundant blood supply, can provide tumor cells with fertile “soil”. The process of liver metastasis is described below. Initially, tumor cells migrate into the circulatory system by invading the surrounding capillaries, venules, lymphatic system, or tissues. After circulation as well as extravasation, tumor cells settle in the liver and eventually form metastases through the process of death, staying in a dormant state or proliferation [4].

Circular RNAs (circRNAs) possess a structure with a covalently closed loop and play a vital role in tumorigenesis by sponging microRNAs (miRNAs), combining with RNA binding proteins (RBPs), or serving as regulators or templates in transcriptional or translational processes, respectively [5–9]. MiRNAs can bind with the region of the target mRNA 3′-UTR, followed by negatively regulating expression [10]. CircRNAs can bind with miRNA response elements (MREs) and inhibit gene activities by sponging miRNA (ceRNA mechanism) [11]. RBPs can mediate multiple metabolic processes of RNAs, such as localization and the formation of ribonucleoprotein complexes [12]. Previous studies have demonstrated that circRNAs can recruit RBPs to change the mRNA stability of downstream genes [13, 14]. It has been reported that circRNAs can mediate chemoradiation resistance as well as multiple malignant processes of cancers, including BC [15, 16]. For example, circTADA2As can impair BC metastasis by mediating miR-203a-3p and its target gene SOCS3 [17]. A novel feedback loop FUS/circEZH2/KLF5/CXCR4 regulates BCLM, which has been previously verified by our team [18]. These results underscore the potential of circRNAs as therapeutic targets for BCLM. Nevertheless, the precise roles and underlying mechanisms of circRNAs in BCLM remain ambiguous and necessitate further inquiry.

We discovered a BCLM-related circRNA, circMYBL2, which could promote BC proliferation and metastasis. The bioinformatic analysis and mechanistic investigations confirmed that circMYBL2 could promote breast carcinogenesis via the upregulation of E2F1 through miR-1205 sponge and the recruitment of eukaryotic translation initiation factor 4A3 (eIF4A3), ultimately culminating in the acceleration of the epithelial-mesenchymal transition (EMT). This study revealed a potentially tumorigenic circRNA in BCLM, which may be a potential therapeutic target and improve the prognosis of patients with BCLM.

## Results

### BCLM tissues and BC cells showed high circMYBL2 expression

**Fig. 1:**
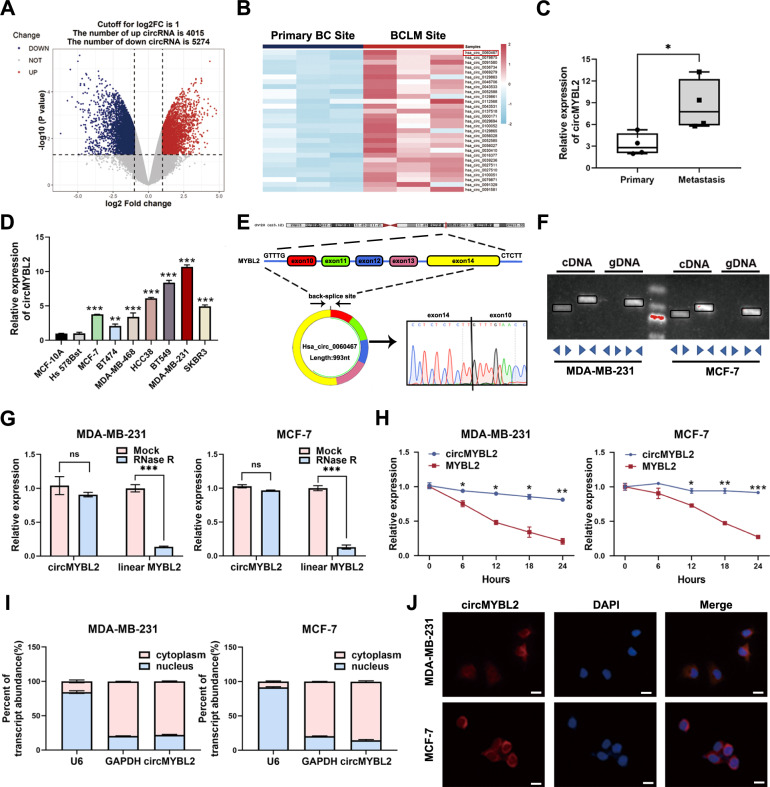
BCLM tissues and BC cells showed high circMYBL2 expression. **A** Using microarray analysis, the expression profile of circRNAs in BCLM tissues was compared to that of primary BC tissues, and visualized through a volcano plot. **B** The heatmap illustrates the top 30 upregulated circRNAs in BCLM tissues compared with primary BC tissues. **C** qRT‒PCR detected the expression of circMYBL2 in four pairs of BCLM and primary BC tissues. The expression of circMYBL2 was normalized to that of GAPDH. **D** qRT‒PCR detected the expression of circMYBL2 in BC cell lines. **E** Schematic illustration showing the structure of circMYBL2 by circularizing exons of the MYBL2 gene. The back-splice site of circMYBL2 was confirmed by Sanger sequencing. **F** PCR assay with divergent and convergent primers showed the amplification of circRNAs from cDNA or gDNA in BC cells in agarose gel electrophoresis. **G** qRT‒PCR detected the expression of circMYBL2 and MYBL2 mRNA in BC cells after RNase R treatment. **H** qRT‒PCR detected the expression of circMYBL2 and MYBL2 mRNA in BC cells after actinomycin D treatment at the indicated times. **I** qRT‒PCR detected the expression of circMYBL2, U6, and GAPDH in the cytoplasm and nucleus of BC cells. CircMYBL2 was normalized to U6 in the nucleus and GAPDH in the cytoplasm. **J** RNA FISH detected the subcellular localization of circMYBL2 in BC cells. Nuclei were stained with DAPI. All data were expressed as the mean ± SD (three independent experiments). *p < 0.05; **p < 0.01; ***p < 0.001.

To screen and characterize aberrantly expressed circRNAs, we used a microarray with three pairs of BCLM tissues (BCLM group, *n* = 3) and primary BC tissues (BC group, *n* = 3). We found that 9289 circRNAs were aberrantly expressed, among which 4015 were elevated and 5274 were reduced in BCLM tissues (Fig. 1A). The first thirty upregulated circRNAs are shown in Fig. 1B. Then, hsa_circ_0060467 (circMYBL2, chr20:42338602–42345122), generated from exon 10 to exon14 of MYBL2 gene, was further explored. We further detected the expression of circMYBL2 in another four pairs of BCLM and primary BC tissues. The statistical analysis was in agreement with microarray results, indicating that circMYBL2 expression was significantly elevated in BCLM tissues (*p* < 0.05, increased in 75% of BCLM tissues, 3/4 × 100% = 75%) (Fig. 1C). Compared with normal breast cells (MCF-10A and Hs 578Bst), BC cells showed high circMYBL2 expression (Fig. 1D). Sanger sequencing confirmed the back-splicing junction of circMYBL2 (Fig. 1E). Linear MYBL2 was detected either in cDNA or gDNA when amplified by corresponding primers, and circMYBL2 was detected only from cDNA (Fig. 1F). In the RNase R digestion assay, circMYBL2 showed more resistance than linear MYBL2 (Fig. 1G). A longer half-life was observed in circMYBL2 but not linear MYBL2 when actinomycin D was added (Fig. 1H). Thus, circMYBL2 has a stable circular structure. Nuclear-cytoplasmic fractionation and RNA FISH analysis showed that circMYBL2 is characterized by cytoplasmic localization (Fig. 1I, J).

### CircMYB2 promoted BC proliferation and migration

**Fig. 2:**
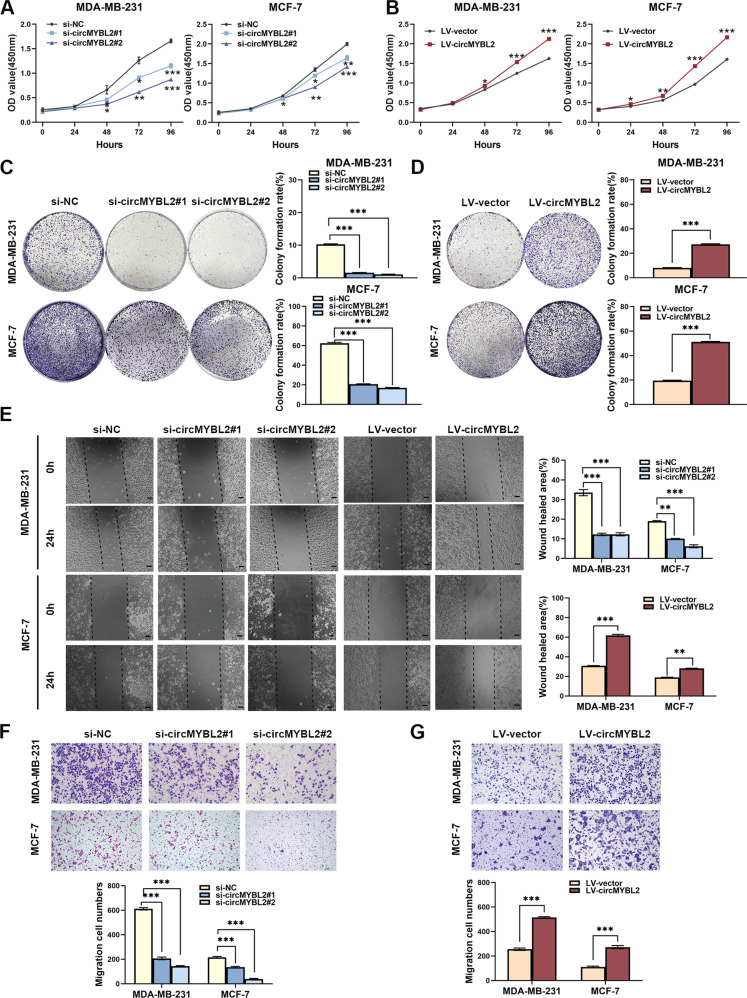
CircMYBL2 promoted BC proliferation and migration. BC cells were transfected with si-circMYBL2 or si-NC and transfected with LV-circMYBL2 or LV-vector via lentivirus plasmids. **A**–**D** For evaluation of the proliferative ability, CCK-8 and colony formation assays were used. **E**–**G** Wound healing and transwell assays were used to evaluate migratory capabilities. The scale bar in wound-healing assays indicates 50 μm; the scale bar in transwell assays indicates 100 μm. All data were expressed as the mean ± SD (three independent experiments). *p < 0.05; **p < 0.01; ***p < 0.001.

Two small interfering RNAs (siRNAs) and lentivirus plasmids were employed to interfere with or stably overexpress circMYBL2 in BC cells (the transfection efficiency was shown in Fig. S1A, B). CCK-8 and colony formation assays confirmed that circMYBL2 knockdown obviously inhibited cell proliferation, whereas proliferative viability could be promoted in the circMYBL2 overexpression group (Fig. 2A–D). Transwell and wound-healing assays clarified that migration was restrained after circMYBL2 inhibition. We obtained the opposite results when circMYBL2 expression was artificially elevated (Fig. 2E–G). In summary, circMYBL2 knockdown negatively impacted BC proliferation and migration.

### CircMYBL2 promoted BC growth and liver metastasis

**Fig. 3:**
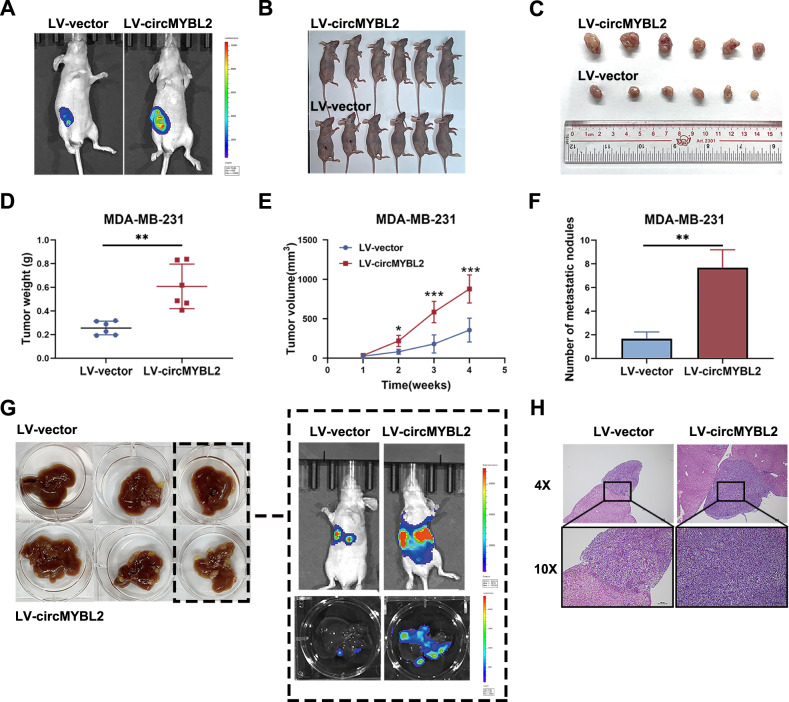
CircMYBL2 promoted BC growth and liver metastasis. MDA-MB-231 cells with stable luciferase expression were infected with LV-circMYBL2 or LV-vector and then injected into BALB/c nude mice. **A** In vivo optical imaging system was used to observe xenograft tumors in the indicated groups. **B**, **C** Representative images of xenograft tumors in the indicated groups. **D** Xenograft tumor weight in the indicated groups. **E** Xenograft tumor volumes were measured and calculated on the indicated days. Tumor volume was measured as (width^2^/2 × length). **F** The number of metastatic nodules in the livers of mice was calculated. **G** Hepatic metastases of BC hemi-spleen models in the indicated groups were collected and monitored by an in vivo optical imaging system. **H** The livers in the indicated groups were stained with H&E. All data were expressed as the mean ± SD. *p < 0.05; **p < 0.01; ***p < 0.001.

Mice (BALB/c nude type) were implanted with MDA-MB-231 cells (stable luciferase expression) infected with LV-circMYBL2 or the corresponding control. Tumors were imaged, extracted, and then weighed after 5 weeks. Tumor volume and weight were markedly elevated when circMYBL2 was overexpressed (Fig. 3A–E). BC hemi-spleen models were constructed successfully with the same cells, and more metastatic nodules appeared at the liver surface in the circMYBL2-overexpressing group (Fig. 3F, G). HE staining confirmed that they were solid tumors (Fig. 3H). Taken together, these experiments verified that circMYBL2 could accelerate growth as well as liver metastasis in BC.

### CircMYBL2 served as a miR-1205 sponge to regulate E2F1 expression

**Fig. 4:**
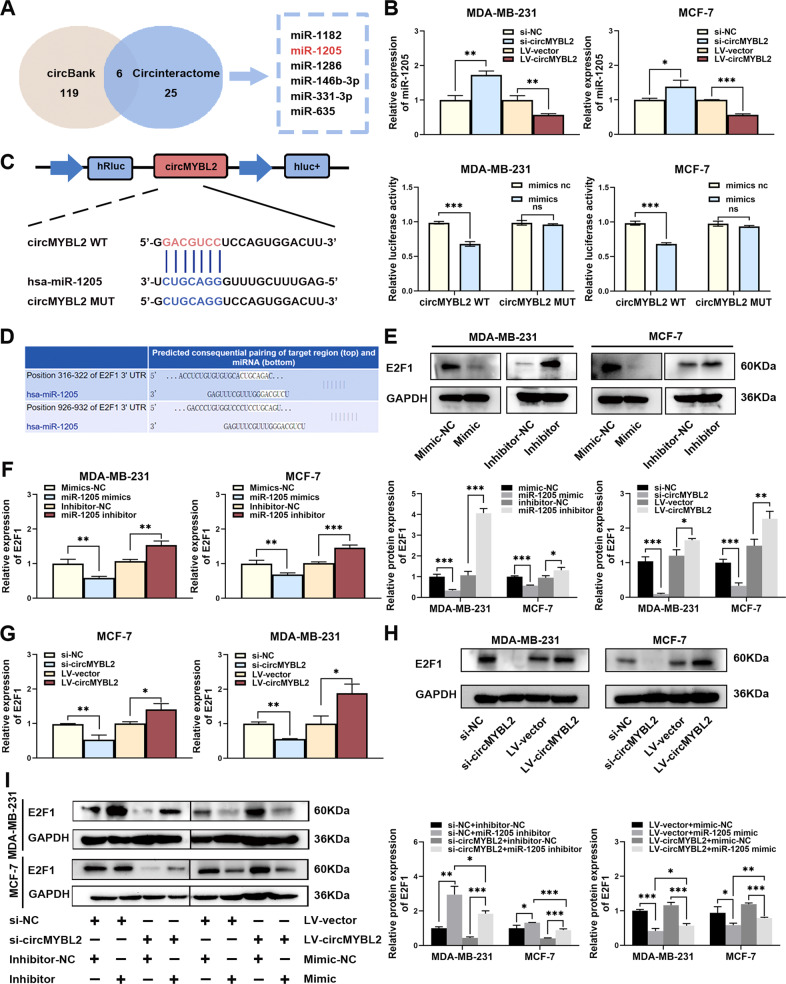
CircMYBL2 served as a miR-1205 sponge to regulate E2F1 expression. **A** Venn diagram of the miRNAs of circMYBL2 based on circBank and CircInteractome. **B** qRT‒PCR detected the expression of miR-1205 in the BC cells transfected with si-circMYBL2 or LV-circMYBL2. **C** Luciferase reporter assays detected the luciferase activities in the BC cells cotransfected with circMYBL2-WT/miR-1205 mimics or circMYBL2-MUT/miR-1205 mimics. **D** The binding sequence between miR-1205 and E2F1 via TargetScan databases. **E**, **F** qRT‒PCR and western blot detected the expression of E2F1 in the BC cells transfected with miR-1205 mimic or inhibitor. **G**, **H** qRT‒PCR and western blot detected the expression of E2F1 in BC cells transfected with si-circMYBL2 or LV-circMYBL2. **I** Western blot analysis of the expression of E2F1 in the BC cells cotransfected with LV-circMYBL2/miR-1205 mimic or si-MYBL2/miR-1205 inhibitor. All data were expressed as the mean ± SD (three independent experiments). *p < 0.05; **p < 0.01; ***p < 0.001.

We predicted candidate-binding miRNAs through the circBank and CircInteractome databases, and six miRNAs were preliminarily screened out (Fig. 4A). MiR-1205 was upregulated in BC cells when circMYBL2 was knocked out and downregulated when overexpressed (Fig. 4B). The luciferase reporter assay exhibited a reduced luciferase intensity in the miR-1205 and wild-type circMYBL2 cotransfected groups, while no significant decrease was observed in the miR-1205 and mutant circMYBL2 cotransfected groups (Fig. 4C). These experiments showed that circMYBL2 may function by sponging miR-1205 in BC cells. According to existing research and predicted results from the TargetScan database, E2F1 was regulated by miR-1205 (Fig. 4D). The results confirmed the regulatory relationship between miR-1205 and E2F1 in BC cells (Fig. 4E, F). We further confirmed whether circMYBL2 could regulate the expression of the miR-1205 target E2F1. E2F1 expression decreased remarkably when circMYBL2 was knocked down (Fig. 4G, H). To confirm whether circMYBL2, miR-1205, and E2F1 could form a regulatory axis, we cotransfected BC cells with si-MYBL2/miR-1205 inhibitors or LV-circMYBL2/miR-1205 mimics. The results demonstrated that circMYBL2 knockdown could suppress E2F1 expression, while the miR-1205 inhibitor partly reversed the expression. Overexpression of circMYBL2 and the miR-1205 mimic produced the opposite results (Fig. 4I). The results showed that circMYBL2 may serve as a ceRNA for miR-1205 and counteract miR-1205-mediated E2F1 inhibition.

### CircMYBL2 recruited eIF4A3 to stabilize E2F1 mRNA

**Fig. 5:**
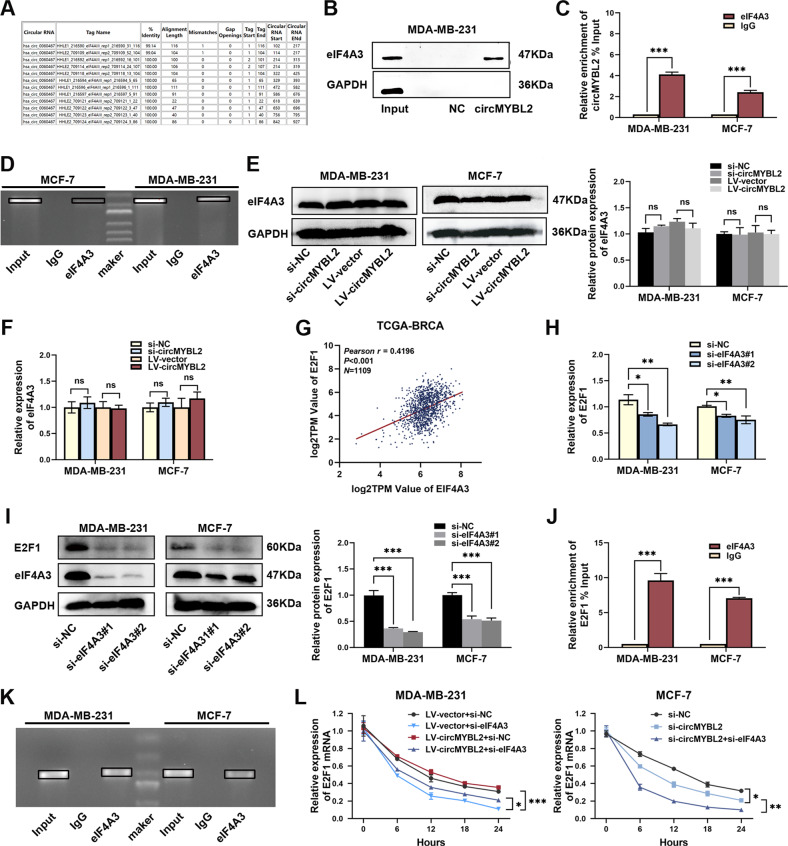
CircMYBL2 recruited eIF4A3 to stabilize E2F1 mRNA. **A** Potential binding site between circMYBL2 and eIF4A3 in CircInteractome. **B** Western blot was used to detect the expression of eIF4A3 pulled down by the circMYBL2 probe or NC via a CHIRP assay. **C**, **D** qRT‒PCR and nucleic acid electrophoresis were used to detect the expression of circMYBL2 pulled down by anti-eIF4A3 or anti-IgG via RIP assay. **E**, **F** Western blot and qRT‒PCR showed the expression of eIF4A3 in the BC cells transfected with si-circMYBL2 or LV-circMYBL2. **G** Pearson’s correlation analysis identified correlations between eIF4A3 and E2F1 in TCGA databases. **H**, **I** qRT‒PCR and western blot showed the expression of E2F1 in the BC cells transfected with si-eIF4A3 or si-NC. **J**, **K** qRT‒PCR and nucleic acid electrophoresis were used to detect the expression of E2F1 mRNA pulled down by anti-eIF4A3 or anti-IgG via RIP assays. **L** qRT‒PCR was used to detect the stability of E2F1 mRNA in BC cells after administration of actinomycin D. All data were expressed as the mean ± SD (three independent experiments). *p < 0.05; **p < 0.01; ***p < 0.001.

According to the prediction of the CircInteractome database, circMYBL2 may have a protein-binding capacity and bind to eIF4A3(Fig. 5A). To confirm this hypothesis, we performed CHIRP and RIP assays. As shown in Fig. 5B, the results of the CHIRP assay followed by western blot validated that eIF4A3 was indeed pulled down by circMYBL2 probes. CircMYBL2 was also enriched primarily in the anti-eIF4A3 group, as validated by the RIP assay (Fig. 5C, D). To determine whether circMYBL2 could regulate eIF4A3 expression, we investigated eIF4A3 mRNA or protein expression with circMYBL2 inhibition or overexpression in BC cells, and no notable changes were detected (Fig. 5E, F). We hypothesized that circMYBL2 may regulate downstream targets by recruiting eIF4A3. The TCGA database showed that E2F1 expression was positively correlated with eIF4A3 in BC tissues (Fig. 5G). We assumed that circMYBL2 may regulate E2F1 by recruiting eIF4A3. Two siRNAs for eIF4A3 inhibition were designed to determine the relationship between eIF4A3 and E2F1 in BC cells (Fig. S1C). E2F1 expression could be inhibited by eIF4A3 silencing (Fig. 5H, I). RIP assays verified the interaction between eIF4A3 and E2F1 mRNA (Fig. 5J, K). eIF4A3 knockdown decreased E2F1 mRNA stability, which could be rescued by circMYBL2 overexpression in BC cells (Fig. 5L). The co-IP assay showed the interaction between E2F1 and eIF4A3 (Fig. S1D). The results suggested that the mechanism between eIF4A3 and E2F1 was worth further analysis.

### Knocking down E2F1 rescued the promotive effect induced by circMYBL2 overexpression in BC

**Fig. 6:**
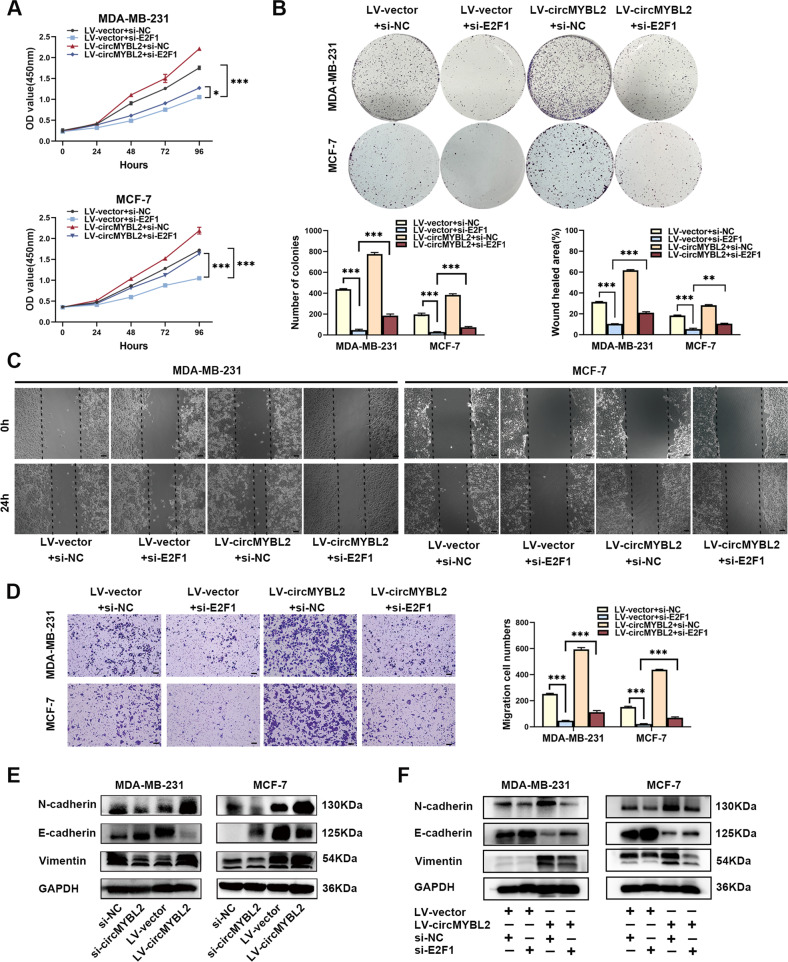
Knocking down E2F1 rescued the promotive effect induced by circMYBL2 overexpression in BC. BC cells were transfected with LV-vector/si-NC, LV-vector/si-E2F1, LV-circMYBL2/si-NC, and LV-circMYBL2/si-E2F1. **A**, **B** For evaluation of the proliferative ability, CCK-8 and colony formation assays were used. **C**, **D** Wound healing and transwell assays were used to evaluate the migratory capabilities of BC cells. The scale bar in wound-healing assays indicates 50 μm; the scale bar in transwell assays indicates 100 μm. **E** Western blot showed the expression of Vimentin, E-Cadherin, and N-Cadherin in the BC cells transfected with si-circMYBL2 or LV-circMYBL2. **F** Western blot showed the expression of Vimentin, E-Cadherin, and N-Cadherin in the BC cells transfected with LV-vector/LV-circMYBL2 and si-NC/si-E2F1. All data were expressed as the mean ± SD (three independent experiments). *p < 0.05; **p < 0.01; ***p < 0.001.

**Fig. 7:**
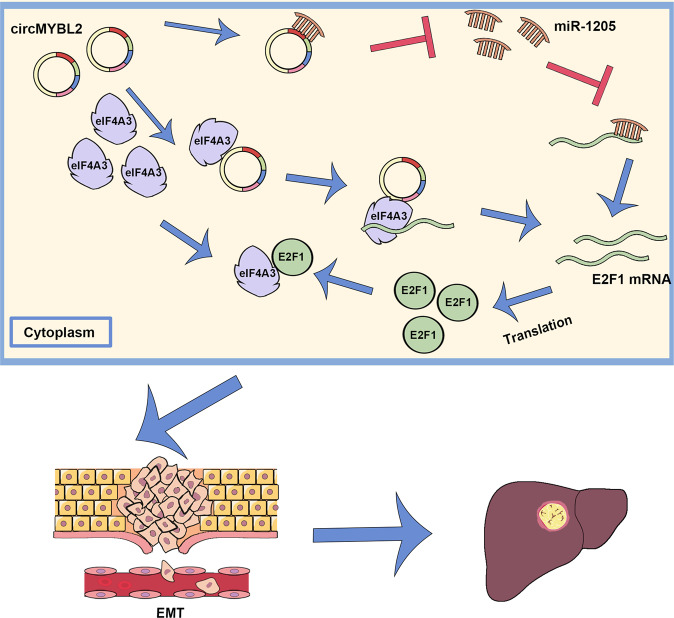
A schematic illustration of the molecular mechanism of circMYBL2 in promoting the development of BC. CircMYBL2 regulates E2F1 expression in BC cells by complexing with eIF4A3 and sponging miR-1205, and eventually activates the process of EMT.

Given these results, rescue assays were used to verify whether circMYBL2 played a promotive role in BC through E2F1. We designed two siRNAs for E2F1 inhibition and chose si-E2F1#2 for subsequent experiments according to the transfection efficiency (Fig. S1E). The CCK-8 and colony formation assays confirmed that E2F1 inhibition reduced the proliferative capacity promoted by LV-circMYBL2 in BC cells (Fig. 6A, B). Transwell and wound-healing assays clarified that E2F1 attenuation reduced the migratory capacity promoted by circMYBL2 overexpression in BC cells (Fig. 6C, D). E2F1 was overexpressed in BC tissues compared with adjacent normal tissues in the UALCAN database (Fig. S1F). High E2F1 expression was significantly correlated with individual cancer stages in BC from UALCAN and GEPIA databases (Fig. S1G, H). Kaplan–Meier plotter database showed that high E2F1 expression was related to poorer OS [hazard ratio (HR) = 1.7, *p* < 0.001] in BC (Fig. S1I). By enhancing E2F1 expression, circMYBL2 could promote BC progression. Moreover, we found that circMYBL2 overexpression reduced E-cadherin while increasing Vimentin and N-cadherin expression in BC cells (Fig. 6E). We overexpressed circMYBL2 and decreased E2F1 together. Western blot analysis indicated that the mesenchymal phenotype induced by circMYBL2 overexpression was inhibited after E2F1 inhibition (Fig. 6F). These findings indicated that circMYBL2 contributed to EMT by elevating E2F1 in BC cells. The mechanism by which circMYBL2 regulates E2F1 by sponging miR-1205 and complexing with eIF4A3 in BC is illustrated in Fig. 7.

## Discussion

Aberrant expression or dysfunction of circRNAs is closely associated with numerous biological and pathological processes, which play an essential role in various tumors, including BC [19, 20]. However, few BCLM-related circRNAs have been fully identified. In a microarray analysis of three pairs of BCLM and primary BC tissues, circMYBL2 emerged as one of the top thirty overexpressed circRNAs. Although prior investigations have identified a cancer-promoting function for circMYBL2 in cervical cancer [21], its specific role and underlying mechanism in BC remain elusive. In this study, we revealed that circMYBL2 was significantly upregulated in BCLM tissues and BC cells, and demonstrated that it promoted BC growth and liver metastasis. We further confirmed that circMYBL2 could regulate E2F1 expression through a miR-1205-mediated ceRNA mechanism and recruit eIF4A3 to stabilize E2F1 mRNA in BC. Eventually, we demonstrated that circMYBL2 could activate the process of EMT by upregulating E2F1 and subsequently contribute to the invasion and metastasis of BC.

It has been reported that miR-1205 restrains the growth of various cancer, such as lung cancer and gastric cancer [22, 23]. Zhong et al. have reported that circRASSF2 regulates the progression of BC through the miR-1205/HOXA1 pathway [24]. To our knowledge, there is only a single study on the role of miR-1205 in BC and the function of miR-1205 remains to be fully elucidated. Previous research has verified that E2F1 serves as a target for miR-1205 in various tumors. Li et al. demonstrated that miR-1205 is downregulated in human laryngeal squamous cell carcinoma (LSCC) and the overexpression of E2F1 can antagonize the suppressive effects of miR-1205 on LSCC. E2F1 could bind to the miR-1205 promoter and transcriptionally inhibit miR-1205 expression [25]. CircCYFIP2 regulates gastric cancer metastasis by mediating the miR-1205/E2F1 axis [23]. CircFN1 mediates sorafenib resistance through the miR-1205/E2F1 axis in hepatocellular carcinoma (HCC) [26]. eIF4A3, one core element of the exon junction complex, is involved in mRNA transport, location, splicing, translation, and degradation [27–29]. It has been suggested that eIF4A3 facilitates the cyclization and biogenesis of circRNAs through its involvement in pre-mRNA splicing events in BC [30, 31]. Studies have revealed that noncoding RNAs, such as circRNAs, can recruit eIF4A3 to regulate its downstream genes post-transcriptionally. For instance, hsa_circ_0068631 could recruit eIF4A3 to make c-Myc mRNA more stable in BC [14]. LncRNA CASC11 regulates E2F1 expression to strengthen E2F1 mRNA stability by recruiting eIF4A3 in HCC [32]. Our study further demonstrated the mechanism of miR-1205 and eIF4A3 in BC.

E2F1, an E2F transcription factor family member, participates in tumor development [33, 34]. According to bioinformatics analysis, E2F1 is remarkably upregulated in BC patients, and elevated expression levels of E2F1 serve as a prognostic indicator for unfavorable outcomes [35]. Overexpression of E2F1 can contribute to BC stemness and tumorigenesis, especially metastasis [36, 37]. Loss of E2F1 significantly impairs the metastatic capacity of HER2/Neu-induced BC [38]. The transcription of PRSS22 initiated by E2F1 promotes BC metastasis by cleaving ANXA1 and activating FPR2/ERK signaling pathway [39]. E2F1-initiated transcription of SEC61G promotes BC metastasis via modulating glycolysis [40]. These results show that E2F1 is a pivotal target or prognostic indicator for BC treatment. Accumulated studies have demonstrated that E2F1 can mediate the EMT process in various cancers, including non-small cell lung carcinoma [41], ovarian cancer [42], clear cell renal cell carcinoma [43], and BC [44]. EMT, where cells lose features of epithelial cells and acquire characteristics of mesenchymal cells, drives dissemination and contributes to metastasis [45, 46]. Our research also revealed that circMYBL2 promoted the EMT process by elevating E2F1 in BC cells. However, the mechanism of how E2F1 mediates EMT in cancer remains largely unknown. Gong et al. observed that E2F1 binds to the promoter region of ZEB1 and enhances the EMT of trophoblast cells by enhancing ZEB1 expression [47]. Based on a considerable body of evidence indicating the association between E2F1 and EMT, we will further investigate the underlying mechanism by which E2F1 regulates EMT in BC. Moreover, circRNAs hold great promise as biomarkers for cancer diagnosis and prognostication, early cancer detection, and as potential therapeutic targets or agents [48]. This study identified circMYBL2 as a potential therapeutic target for patients with BCLM.

One limitation of the study is the absence of verification of the correlation between circMYBL2 and the prognosis of BC patients due to the restriction of existing databases and available specimens. Our group will further enhance the collection of relevant specimens. In addition, an inadequacy exists concerning the absence of a conclusive mechanism by which circMYBL2 stimulates hepatic metastasis of BC, as opposed to other anatomical regions of the body. Although we found that circMYBL2 can promote BCLM through the EMT pathway, it should be noted that EMT constitutes the principal requisite for tumor metastasis. Therefore, it warrants further inquiry into whether circMYBL2 can induce liver metastasis of BC through other exclusive pathways.

In conclusion, circMYBL2 could promote tumorigenesis and aggressiveness of BC through the circMYBL2/miR-1205/E2F1 axis and circMYBL2/eIF4A3/E2F1 axis. Our findings revealed an oncogenic circRNA in BCLM, which could be a potential therapeutic target for patients with BCLM.

## Materials and methods

### Tissues and ethical authorization

All BC samples were acquired from Sun Yat-Sen University Cancer Center, Guangzhou, China. This research was permitted by the Ethics Committee of our current unit. Informed consent was provided and signed by all patients prior to their inclusion.

### Cell culture and transfection

The ATCC Cell Biology Collection (USA) provided MCF-7, BT-474, MDA-MB-468, HCC38, MDA-MB-231, BT-549, SKBR-3, MCF-10A, and Hs 578Bst cells. All cells were cultured according to the manufacturer’s guidelines. Hanheng Biotech (Shanghai, China) constructed the lentivirus-based vectors and GenePharma (Shanghai, China) constructed the siRNAs, miRNA mimics, or inhibitors. Table S1 shows the sequences of all siRNAs. Detailed procedures of cell transfection were provided in Supplementary Material and Methods.

### In vitro experiments

Full detailed procedures of quantitative real-time PCR (qRT-PCR), actinomycin D treatment, RNase R assay, subcellular fractionation, fluorescence in situ hybridization (FISH), cell viability and migration assay, dual-luciferase reporter assay, RNA immunoprecipitation (RIP), chromatin Isolation by RNA Purification (CHIRP) assay, western blot, and immunoprecipitation (IP) were provided in Supplementary Material and Methods.

### Xenograft experiments

In vivo experiments were performed following institutional and international guidelines and regulations. Detailed procedures were provided in Supplementary Material and Methods.

### Bioinformatics and statistical analysis

E2F1 and eIF4A3 mRNA expression in BC patients were acquired from the TCGA database. MiRNAs that may act as sponges of circRNAs were predicted at the circBank and CircInteractome websites. TargetScan was adopted to predict possible binding sites between miRNAs and target genes. The StarBase and TCGA databases were adopted for candidate genes exploring coexpression networks. Proteins binding to circRNAs were predicted at the CircInteractome website. The Kaplan–Meier plotter was adopted to predict the overall survival (OS) of BC patients. UALCAN and GEPIA databases were adopted for E2F1 expression in different cancer stages. GraphPad Prism 8.0 software was implemented for data analyses. The two-tailed Student’s *t*-test, Pearson’s correlation, and one-way analysis of variance were employed in data analysis. All data were given as the mean ± SD. When *p* < 0.05, statistical significance was established.

## Supplementary information

Supplementary Materials and Methods.

Supplementary Figure Legend

Supplementary Table

Western blots

Supplementary Figure1

## Data Availability

The data supporting the findings of this study are available within the article and its supplementary materials.
