# Supplementary material for: Hsa_circ_0060467 promotes breast cancer liver metastasis by complexing with eIF4A3 and sponging miR-1205
Source: Cell Death Discov. 2023 May 9;9:153. doi: 10.1038/s41420-023-01448-4 (PMC10169853; doi:10.1038/s41420-023-01448-4)
Supplement: Supplementary file 1 — Supplementary Materials and Methods. [file 41420_2023_1448_MOESM1_ESM.docx]

**Supplementary Materials and Methods**

**Cell transfection**

GenePharma (Shanghai, China) constructed the siRNAs of circMYBL2, eIF4A3, and E2F1, miR-1205 mimics, and miR-1205 inhibitors. All siRNA sequences were shown in Table S1. Exponentially growing culture of BC cells were seeded in a six-well plate. Cells were transfected at 70–80% monolayer confluency. 1.0×10^6^ cells were transfected with 2 μg of siRNAs using a Lipofectamine -3000 Transfection Kit (Thermo Fisher Scientific) following the standard protocol. The cells were incubated for 6 h with a transfection mixture. The medium was then replaced with fresh DMEM supplemented with 10% FBS, and the cells were incubated for another 48 h.

Hanheng Biotech (Shanghai, China) constructed the cricMYBL2 overexpression vector by cloning the full length of human circMYBL2 into a PLCDH-ciR vector and provided lentiviral supernatant with a density of 1× 10^8^ TU/ml. Exponentially growing culture of BC cells were seeded in a six-well plate. Cells were transfected at 70–80% monolayer confluency. The cells were incubated for 12 h with lentiviral supernatant and 10 μg/mL polybrene following the manufacturer’ instructions. The medium was then replaced with fresh DMEM supplemented with 10% FBS, and the cells were incubated for another 48-72 h. Finally, the cells were maintained in medium containing puromycin and effective clones were selected.

**RNA isolation and quantitative real-time PCR analysis (qRT‒PCR)**

Total RNA was extracted by the RNA-Quick Purification kit (YiShan Biotech, China). The reverse transcription of circRNAs and mRNA was performed using the PrimeScript™ Master Mix (TaKaRa, Shiga, Japan) and subsequent quantification was conducted via qRT-PCR using the SYBR Green Master Mix (TaKaRa, Shiga, Japan). For miRNA quantification, cDNA was produced by the PrimeScript™ RT reagent kit (TaKaRa, Shiga, Japan) and the SYBR Green Master Mix (TaKaRa, Shiga, Japan) was utilized in subsequent quantification. The thermal protocol for qRT-PCR reaction was as follows: initial denaturation at 95°C for 5 minutes, followed by 40 cycles of denaturation at 95°C for 15 seconds, annealing at 60°C for 30 seconds, and extension at 72°C for 30 seconds. The fluorescence signal was measured at the end of each extension step. GAPDH and U6 were served as the control, respectively. Primers adopted hereby were shown in Table S2.

**Actinomycin D treatment**

Actinomycin D (2 μg/ml, Glpbio, USA) was added to BC cells culture and total RNA was obtained at different time points (beginning at 0, 6, 12, 18 and 24 h), followed by expression detection.

**RNase R assay**

Total RNA was adopted for the RNase R assay (4 U/μg, Beyotime, China). After 30 min of incubation time, treated RNA were then transcribed into cDNA. The expression of circMYBL2 and linear MYBL2 mRNA were detected.

**Subcellular fractionation**

Nuclear and cytoplasmic extraction reagents (Thermo Fisher Scientific, USA) were used to separate the subcellular fractions from BC cells following the standard protocol. U6 and GAPDH served as the nuclear and cytoplasmic controls, respectively.

**Fluorescence in situ hybridization (FISH)**

GenePharma (China) provided a fluorescence in situ hybridization kit and circMYBL2 probes. BC cells were incubated with the circMYBL2 probe on glass cover slips in 24-well plates overnight according to the manufacturer’s protocol. DAPI was used to stain the nuclei. Fluorescence images were assessed by microscope (Olympus BX53, Japan).

**Cell** **proliferation assay**

BC cells were transferred into plates (96-well, 10^3^ each) after transfection. Forty-eight hours later, CCK-8 (GlpBio, USA) solution was added to plates. The absorbance at 490 nM was measured 2 h later.

**Colony formation assay**

BC cells were transferred to plates (6-well plates, 10^3^ each). Fourteen days later, methanol was used to fix the colonies, followed by crystal violet (0.1%) staining. The stained colonies were then assessed and counted under the microscope.

**Wound-healing assay**

BC cells were transferred to plates (6-well, 10^3^ each) after transfection. Pipette tips (200 μL) were adapted to make the linear wound. Migration was then assessed and counted under the microscope at different time points (beginning at 0, 12, and 24 h).

**Transwell assay**

Cells were digested 24 hours after transfection and then seeded into the upper chambers (3*10^4^ cells/well). The upper cells were incubated without FBS, and 20% FBS was added to the lower cells. Twenty-four hours later, 0.1% crystal violet was used to stain the cells in the upper chambers after methanol fixation, and the stained cells were counted under microscopy.

**Dual-luciferase reporter assay**

Umine-bio (China) provided the plasmids (wild/mut type sequences of circMYBL2 and luciferase reporter contained). The above wild-type or mutant reporter vectors as well as the mimics of miR-1205 were cotransfected to BC cells. Forty-eight hours after transfection, Firefly and Renilla luciferase activities were assessed by Firefly and Renilla Luciferase Kit (Yeasen, China) according to the manufacturer’s protocol.

**RNA immunoprecipitation (RIP) assay**

An RNA Immunoprecipitation Kit (BersinBio, China) was used in this assay. According to the manufacturer’s protocol, lysed BC cells were incubated in RIP buffer with anti-eIF4A3 antibodies or IgG-conjugated magnetic beads. After proteinase K incubation, immunoprecipitated RNAs were isolated. The expression of circMYBL2 and E2F1 in the precipitants was assessed by qRT‒PCR.

**Chromatin Isolation by RNA Purification (CHIRP) assay**

A Chromatin Isolation by RNA Purification Kit (BersinBio, China) was adopted to this assay. RiboBio (China) constructed and offered a biotin-labeled probe that targeted the circMYBL2 junction site. Following the manufacturer’s protocol, specific probes, cell lysates and magnetic beads (streptavidin conjugated) were adopted to isolate the RNA‒protein complexes. eIF4A3 expression was ultimately assessed by western blot assays.

**Western blot**

PMSF and RIPA lysis were used for protein extraction, followed by protein separation, transfer and primary antibody incubation including eIF4A3 (Abcam, USA), E2F1 (Proteintech, USA), EMT-related primary antibodies such as Vimentin, E-cadherin, N-cadherin (Proteintech, USA), and GAPDH (Proteintech, USA) at a density of 1:10^3^. Secondary antibodies were subsequently utilized. Finally, the target protein was visualized and quantified.

**Immunoprecipitation (IP)**

BC cells were collected and lysed. The 5% supernatant [consist](https://cn.bing.com/dict/search?q=consist&FORM=BDVSP6&cc=cn)ed [of](https://cn.bing.com/dict/search?q=of&FORM=BDVSP6&cc=cn) the input group. Then, the instructions were followed to incubate the remaining supernatant overnight (primary antibody or IgG). Subsequently, the supernatants and protein A/G-magnification beads (MedChemExpress, USA) were incubated. Two hours later, the beads were washed 3 times. Then, western blot was adopted for immunoprecipitated protein detection.

**Xenograft experiments**

The mice (BALB/c nude type, female, 4-week-old) were implanted with MDA-MB-231 cells (2x10^6^, stable luciferase expression) infected with LV-circMYBL2 or the corresponding control. After 35 days, 100 µl of D-luciferin was injected into each mouse and images were taken and analyzed. Tumors were extracted for weight and volume measurements.

For establishment of the hepatic metastases of BC hemi spleen models, the mice (BALB/c nude type, female, 6-week-old) were injected with the above cells at a density of 1x10^6^ through spleens. Eight weeks later, 100 µl of D-luciferin was injected into each mouse and photographed. Liver tissues were extracted and paraffin-embedded for H&E detection. All experimental and animal care procedures followed the rules from our organization.
