# Supplementary material for: Hsa_circ_0060467 promotes breast cancer liver metastasis by complexing with eIF4A3 and sponging miR-1205
Source: Cell Death Discov. 2023 May 9;9:153. doi: 10.1038/s41420-023-01448-4 (PMC10169853; doi:10.1038/s41420-023-01448-4)
Supplement: Supplementary file 2 — Supplementary Figure Legend [file 41420_2023_1448_MOESM2_ESM.doc]

**Electronic Supplementary Material (ESM)**

Fig. S1 A-B CircMYBL2 expression after siRNA and lentivirus plasmid transfection in BC cells. C eIF4A3 expression after siRNA transfection in BC cells. D Protein validation of the binding relationship between eIF4A3 and E2F1 after the co-IP assay. E E2F1 expression in BC cells transfected with siRNAs. F E2F1 expression in BC tissues compared with adjacent normal tissues in UALCAN database. G-H GEPIA and UALCAN databases showed the expression of E2F1 in individual cancer stages of BC. I Kaplan-Meier plotter database showed the relationship between E2F1 and OS.
