# Supplementary material for: Hsa_circ_0060467 promotes breast cancer liver metastasis by complexing with eIF4A3 and sponging miR-1205
Source: Cell Death Discov. 2023 May 9;9:153. doi: 10.1038/s41420-023-01448-4 (PMC10169853; doi:10.1038/s41420-023-01448-4)
Supplement: Supplementary file 3 — Supplementary Table [file 41420_2023_1448_MOESM3_ESM.doc]

**Table S1. siRNA sequences**

| **Primer** | **Forward (5’-3’)** | **Reverse (5’-3’)** |
| --- | --- | --- |
| si-circMYBL2#1 | CUCUCUCUUGUUUGUAACCTT | GGUUACAAACAAGAGAGAGTT |
| si-circMYBL2#2 | CUUGUUUGUAACCCCAGAUTT | AUCUGGGGUUACAAACAAGAG |
| si-E2F1#1 | CCUGGAAACUGACCAUCAGTT | CUGAUGGUCAGUUUCCAGGTT |
| si-E2F1#2 | GACCACCUGAUGAAUAUCUTT | AGAUAUUCAUCAGGUGGUCTT |
| si-eIF4A3#1 | AAGCAGCAGAUCAGUGGGAUGAGTT | CUCAUCCCACUGAUCUGCUGCUUTT |
| si-eIF4A3#2 | AAUCCAGCAACGAGCAAUCTT | GAUUGCUCGUUGCUGGAUUGC |
|  |  |  |

**Table S2. qRT-PCR Primers**

| **Primer** | **Forward (5’-3’)** | **Reverse (5’-3’)** |
| --- | --- | --- |
| CircMYBL2  MYBL2 | GCCTCTCTCTTGTTTGTAACCCC  CCGGAGCAGAGGGATAGCA | TCAGAACGCAGCACCTCCTT  CAGTGCGGTTAGGGAAGTGG |
| eIF4A3 | CGCGGACTCTGACATATGGCGACCACGGCCACGATG | TCCCGCAGGCCCATGGTGTCG |
| E2F1 | CCGTGGACTCTTCGGAGAAC | ATCCCACCTACGGTCTCCTC |
| GAPDH | GAAGGTGAAGGTCGGAGTC | GAAGATGGTGATGGGATTTC |
| U6 | CGAGCACAGAATCGCTTCA | CTCGCTTCGGCAGCACATAT |
