# Supplementary material for: Hsa_circ_0060467 promotes breast cancer liver metastasis by complexing with eIF4A3 and sponging miR-1205
Source: Cell Death Discov. 2023 May 9;9:153. doi: 10.1038/s41420-023-01448-4 (PMC10169853; doi:10.1038/s41420-023-01448-4)
Supplement: Supplementary file 4 — Western blots [file 41420_2023_1448_MOESM4_ESM.pdf]

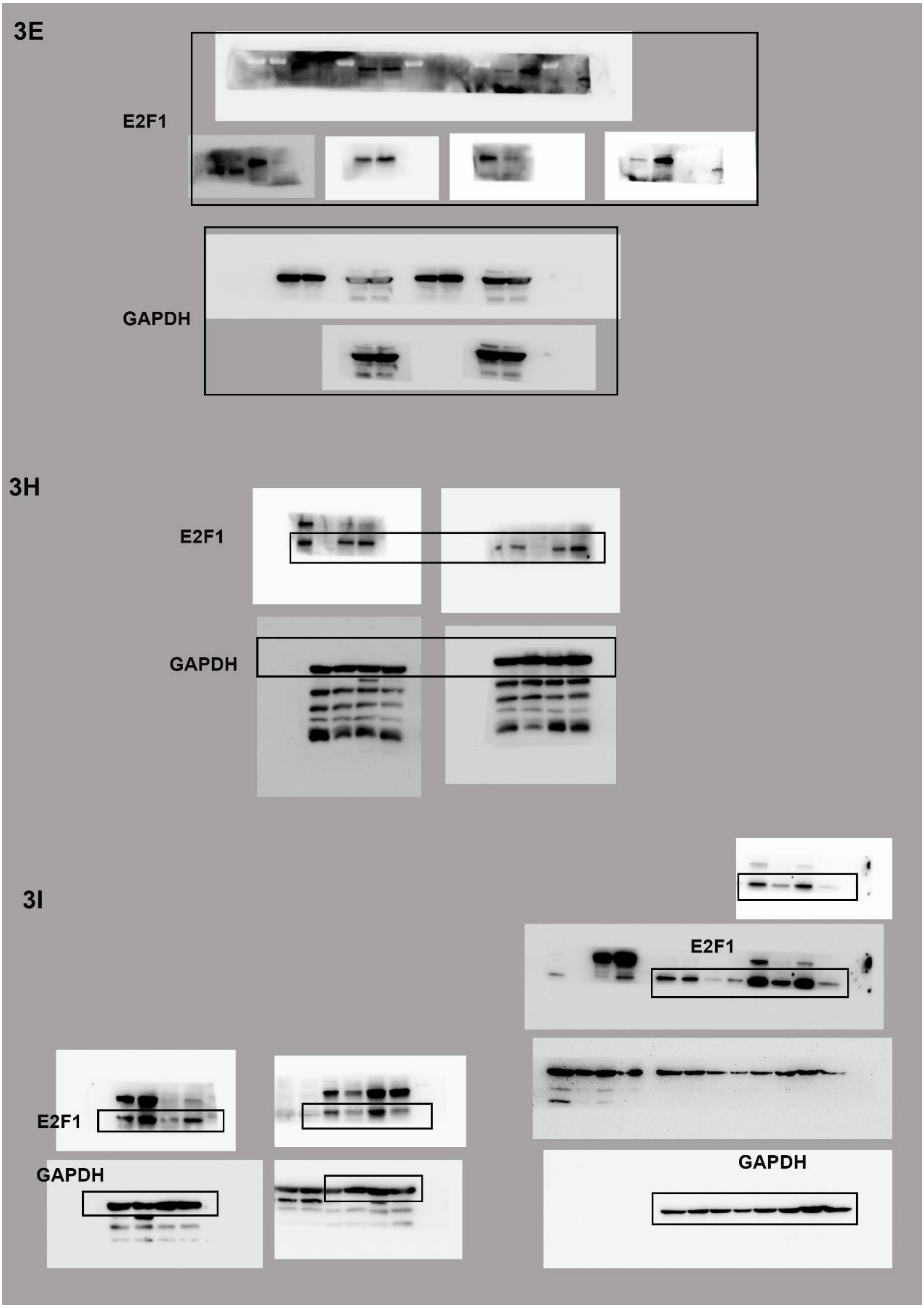
3E
E2F1
GAPDH
3H
E2F1
GAPDH
3I
E2F1
E2F1
GAPDH
GAPDH

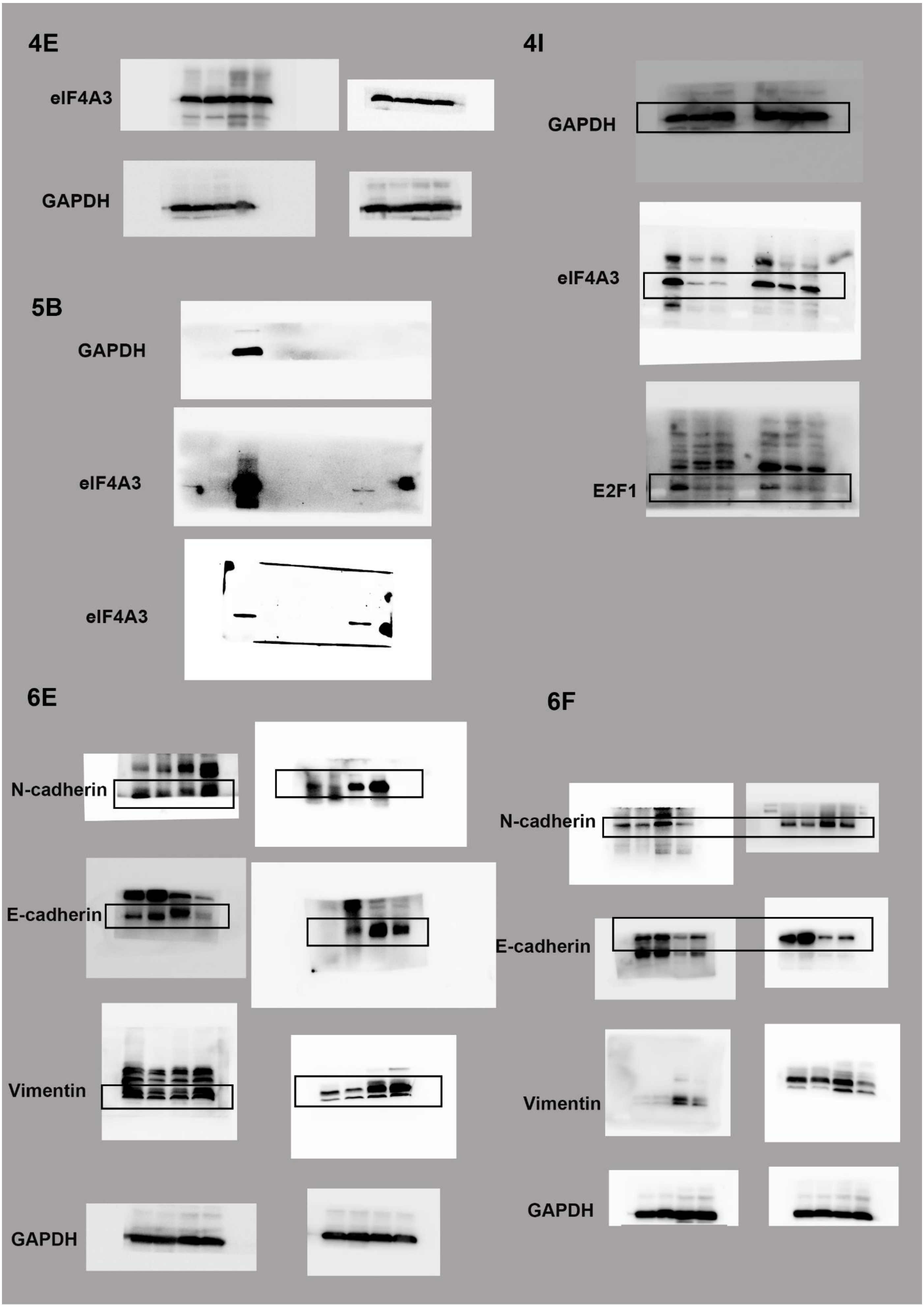
4E
eIF4A3
GAPDH
4I
GAPDH
eIF4A3
E2F1
5B
GAPDH
eIF4A3
eIF4A3
6E
N-cadherin
E-cadherin
Vimentin
GAPDH
6F
N-cadherin
E-cadherin
Vimentin
GAPDH
