# Supplementary figures and images for: Hsa_circ_0060467 promotes breast cancer liver metastasis by complexing with eIF4A3 and sponging miR-1205
Source: Cell Death Discov. 2023 May 9;9:153. doi: 10.1038/s41420-023-01448-4 (PMC10169853; doi:10.1038/s41420-023-01448-4)

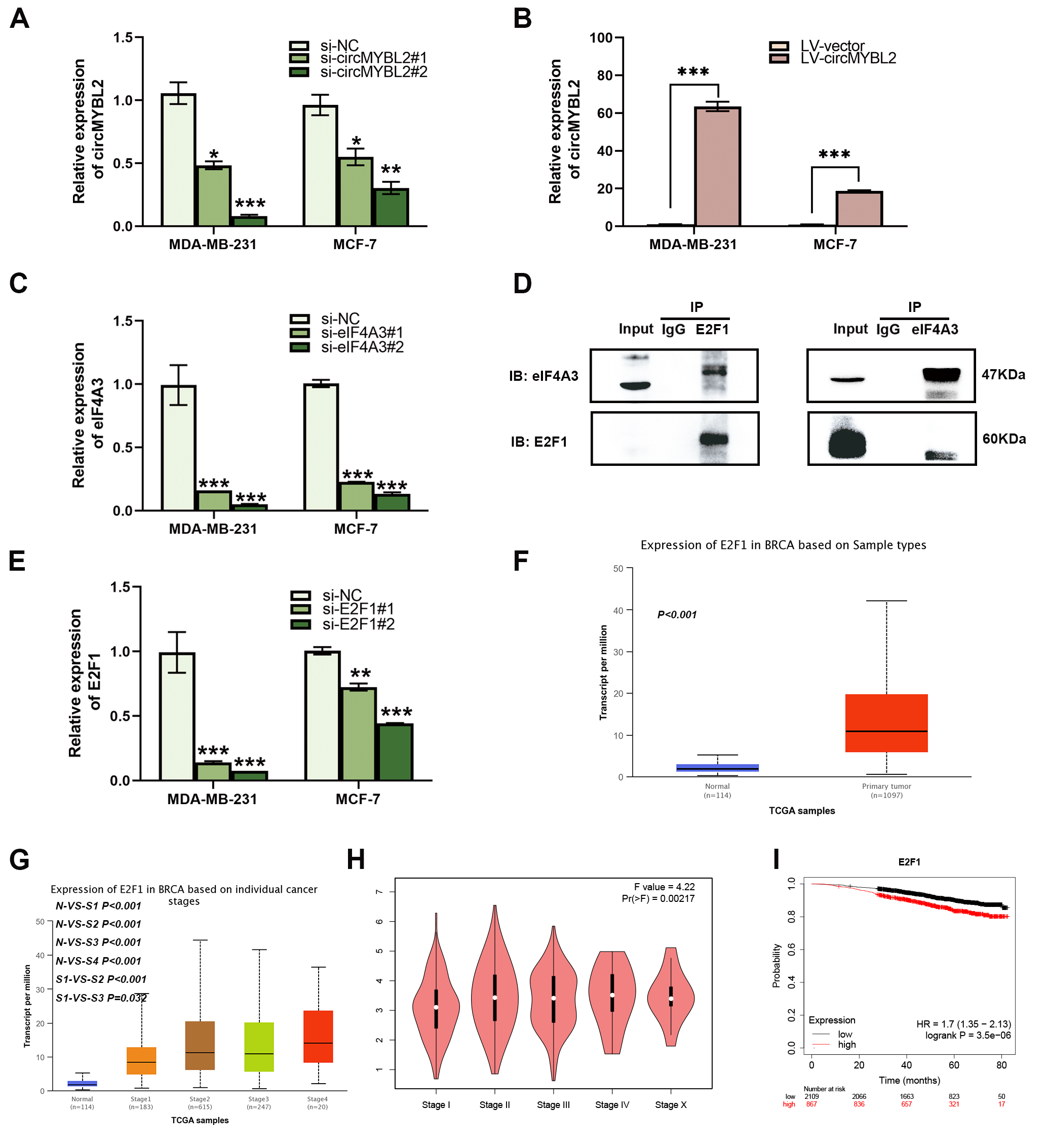

Supplement: Supplementary file 5 — Supplementary Figure1 [file 41420_2023_1448_MOESM5_ESM.tif]
